# Fourier-Transform Infra-Red Microspectroscopy Can Accurately Diagnose Colitis and Assess Severity of Inflammation

**DOI:** 10.3390/ijms23052849

**Published:** 2022-03-05

**Authors:** Charlotte Keung, Philip Heraud, Nathan Kuk, Rebecca Lim, William Sievert, Gregory Moore, Bayden Wood

**Affiliations:** 1 School of Clinical Sciences, Monash University, Melbourne, VIC 3168, Australia; nathan.kuk@monashhealth.org (N.K.); rebecca.lim@hudson.org.au (R.L.); william.sievert@monash.edu (W.S.); gregory.moore@monash.edu (G.M.); 2 Department of Gastroenterology, Monash Health, Melbourne, VIC 3168, Australia; 3 Monash Centre for Biospectroscopy, Monash University, Melbourne, VIC 3800, Australia; prheraud@gmail.com (P.H.); bayden.wood@monash.edu (B.W.); 4 The Ritchie Centre, Hudson Institute of Medical Research, Melbourne, VIC 3168, Australia

**Keywords:** vibrational spectroscopy, infra-red spectroscopy, colitis, inflammatory bowel disease

## Abstract

The diagnosis and management of inflammatory bowel disease relies on histological assessment, which is costly, subjective, and lacks utility for point-of-care diagnosis. Fourier-transform infra-red spectroscopy provides rapid, non-destructive, reproducible, and automatable label-free biochemical imaging of tissue for diagnostic purposes. This study characterises colitis using spectroscopy, discriminates colitis from healthy tissue, and classifies inflammation severity. Hyperspectral images were obtained from fixed intestinal sections of a murine colitis model treated with cell therapy to improve inflammation. Multivariate analyses and classification modelling were performed using supervised and unsupervised machine-learning algorithms. Quantitative analysis of severe colitis showed increased protein, collagen, and nucleic acids, but reduced glycogen when compared with normal tissue. A partial least squares discriminant analysis model, including spectra from all intestinal layers, classified normal colon and severe colitis with a sensitivity of 91.4% and a specificity of 93.3%. Colitis severity was classified by a stacked ensemble model yielding an average area under the receiver operating characteristic curve of 0.95, 0.88, 0.79, and 0.85 for controls, mild, moderate, and severe colitis, respectively. Infra-red spectroscopy can detect unique biochemical features of intestinal inflammation and accurately classify normal and inflamed tissue and quantify the severity of inflammation. This is a promising alternative to histological assessment.

## 1. Introduction

### 1.1. Role of Histology in IBD

Inflammatory bowel disease (IBD) is a chronic inflammatory immune-mediated condition with globally increasing prevalence with time [1]. Disease onset is common in young adulthood and currently it is the fifth leading cause of years lived with disability amongst digestive diseases [2]. Histological assessment of tissue obtained from surgical specimens or endoscopic biopsies remains a cornerstone for the diagnosis and management of IBD. The European Crohn’s and Colitis Organisation and British Society of Gastroenterology consensus guidelines continue to recognise histology, from either endoscopic biopsies or surgical resections, as a key factor in IBD diagnosis. Endoscopic biopsy for histological assessment is the gold standard of care for ongoing activity and dysplasia surveillance [3,4]. With increased biological and small molecule therapies emerging for IBD management, treatment targets, particularly in ulcerative colitis, are moving towards histological remission, placing even greater reliance on histological assessment [5].

However, histological evaluation of IBD for diagnosis, activity, and dysplasia assessment is affected by multiple issues, including the lack of standardised and/or validated diagnostic criteria, inter- and intra-observer variation and uncertainty regarding clinical correlation [6]. Furthermore, the time taken for specimen processing, staining, and assessment precludes its use for point-of-care diagnosis. Moreover, in addition to accumulative cost with multiple specimens obtained during the patient’s lifetime, histological findings do not predict disease course or treatment response.

### 1.2. Application of Biospectroscopy Technologies in IBD

Biospectroscopy allows for non-destructive, label-free biochemical analysis of biological specimens through the measurement of absorbance from molecular vibrations. Fourier transform infra-red (FTIR) spectroscopy uses an infra-red energy source to atomically displace molecular bonds with quantitative analysis of the absorbance intensities. The biological fingerprint is located at 1800–800 cm^−1^ and contains the most important infra-red spectral regions of interest for biological specimens, which have been well-characterised. Additionally, this can be coupled with a focal plane array detector and microscope, allowing for collection of hyperspectral images that include both spectral, morphological, and spatial information, providing an ideal method to study tissue specimens. Spectral data are then routinely analysed via multivariate analyses that incorporate machine-learning algorithms for exploratory analyses as well as classification models, ultimately to create reproducible, automatable, and standardised results [7].

Published studies using this technology for intestinal inflammation are limited. Human studies using FTIR spectroscopy for IBD research have largely only investigated colitis as a secondary comparator to colorectal cancer. Differences in the biochemical composition of inflamed bowel compared with cancer [8,9,10] were demonstrated, with models showing predictive capacity for cancer diagnosis. Studies applying FTIR to animal models of colitis are also limited but have demonstrated that biochemical signatures can accurately classify colitis in normal tissue [11] and serum [12,13], with a potential for monitoring treatment response [14]. Most of these studies with intestinal tissue, however, do not distinguish between the layers of the bowel or only include the mucosa with limited sampling of random spectra only.

### 1.3. Aim and Hypothesis

The aim of this study is to characterise the tissue biochemistry of intestinal inflammation using FTIR microspectroscopy and demonstrate that the spectra can be used to accurately classify healthy tissue from colitis and further assess the severity of inflammation. We hypothesise that the biochemical composition of colitis is distinct and can be quantitatively assessed using this technique. To our knowledge, this is the first study using FTIR spectroscopy to assess the severity of intestinal inflammation.

## 2. Results

### 2.1. General Results

**Figure 1:**
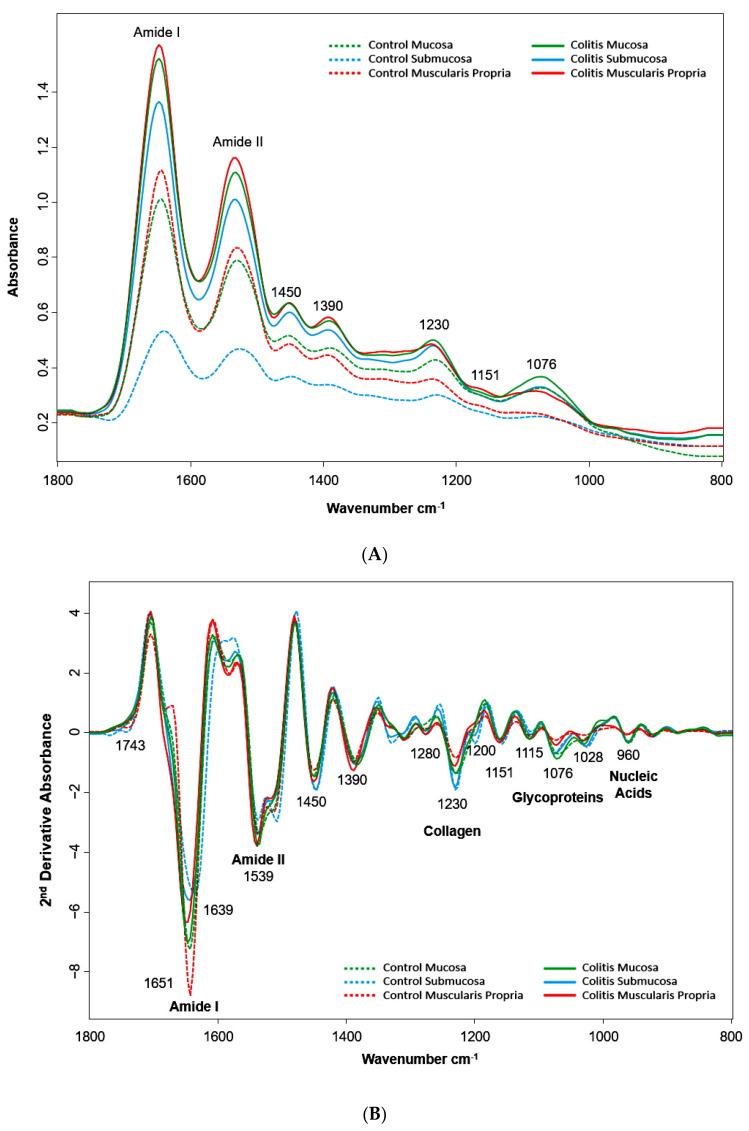
Representation of average spectra obtained from healthy tissue compared with severe colitis. (**A**) Comparison of average original spectra for controls and severe colitis across all layers of the bowel wall. (**B**) Comparison of average second derivative spectra for controls and severe colitis across all layers of the bowel wall.

Over 500,000 spectra were collected from 99 sections of colon from 30 mice, creating a large dataset which required data reduction, as described in the methodology. The “biological fingerprint” region from 1800–800 cm^−1^ was used for analysis and the average spectra for each intestinal layer are shown below in Figure 1.

We found that there was, overall, a higher absorbance intensity in the mean (baseline-corrected) colitis spectra compared with control mice. The main protein peaks (amide I and II located at 1700–1600 cm^−1^ and 1580–1510 cm^−1^, respectively) were the greatest in the muscularis propria (MP) when comparing bowel wall layers amongst control and severe colitis groups (Figure 1A).

Using second derivative spectra (Figure 1B), where the maxima have now become minima, there were clear differences between the colitis and control groups, as well as amongst the different layers of the bowel. Table 1 lists the infra-red absorbance band assignments for the wavenumber values in the spectra of intestinal tissue corresponding to their biochemical composition.

The minima at the large amide I protein band shows that the protein configurations in the mucosa (MC) and MP in both control and colitis are predominantly of α-helical secondary structure (1646–1640 cm^−1^), whereas the main amide I protein conformation for the submucosa (SubMC) has a shifted minimum located at 1635 cm^−1^ representing the β-sheet protein conformation. The “collagen triplet” [20] can be clearly identified by minima located on second derivative spectra at approximately 1280 cm^−1^, 1230 cm^−1^, and 1200 cm^−1^, although it should be noted that the 1230 cm^−1^ band also has a contribution from an asymmetric phosphodiester stretch. The absorbance is greatest at the “collagen triplet” for the SubMC, as the most abundant extra-cellular matrix is collagen. The glycoprotein band at 1076 cm^−1^ has the highest absorbance in the MC spectra, likely due to the presence of mucous produced from goblet cells (although nucleic acids also contribute to the absorbance intensity at this wavenumber value). There are significantly fewer nucleic acids in the MP layer compared with the other layers, as demonstrated by the band at 960 cm^−1^ which is assigned to the C-C and C-H deoxyribose in DNA [21].

Overall, there also appears to be greater variation amongst the spectra comparing the different bowel wall layers (containing different structural elements) than between the control and diseased groups.

### 2.2. Hyperspectral Image Analysis Results of Intestinal Mucosa

#### 2.2.1. Biochemical Composition

**Figure 2:**
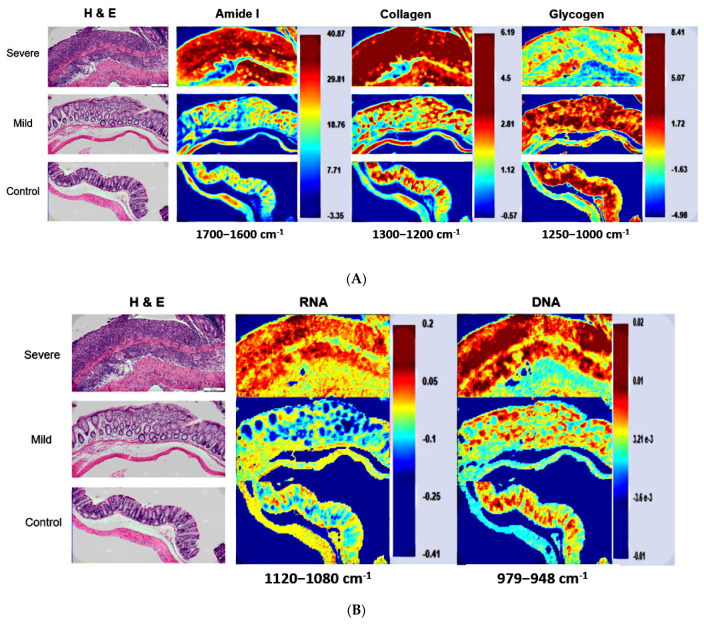
Comparison of the integrated areas under regions of interest in the spectra in healthy tissue, mild, and severe colitis. (**A**) Quantitative representation of protein, collagen, and glycogen. (**B**) Quantitative representation of RNA and DNA.

**Figure 3:**
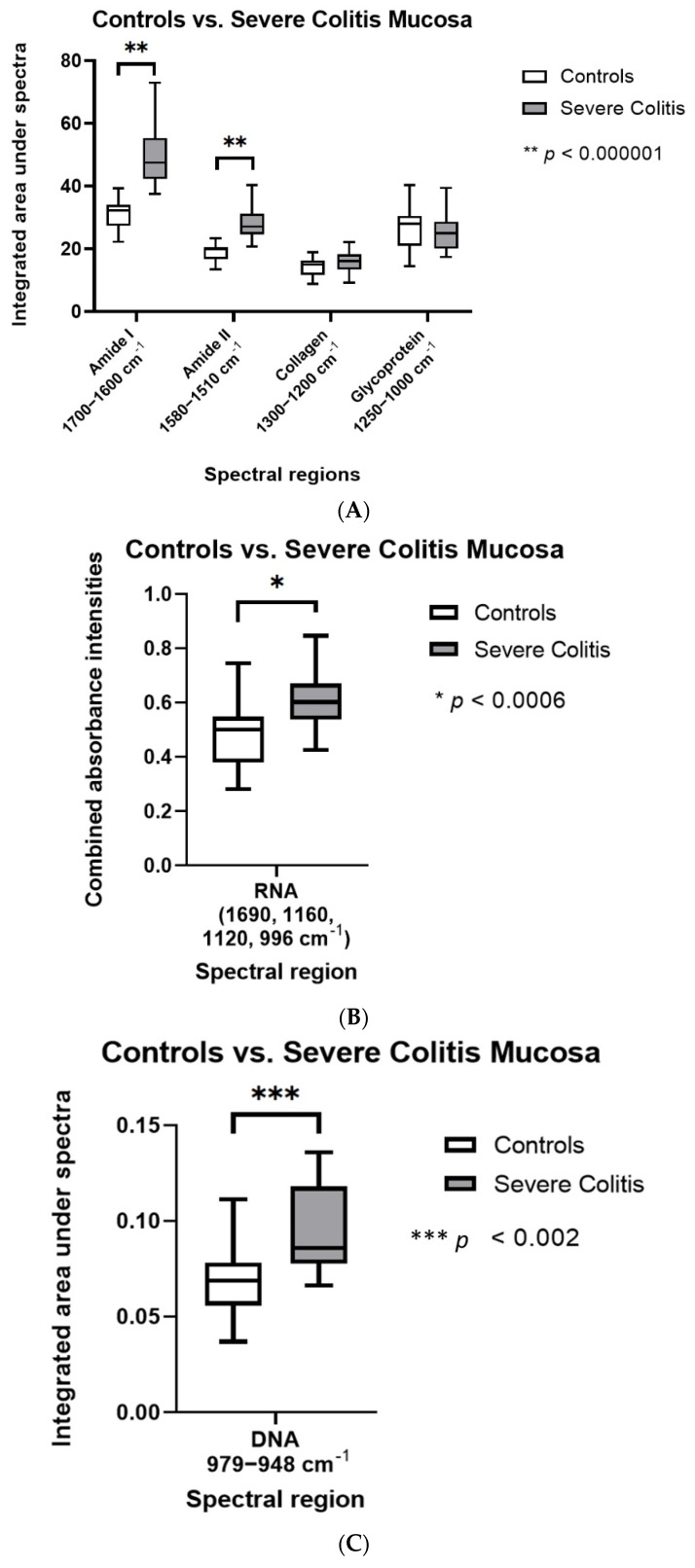
Quantitative comparison of the biochemical composition between the mucosa of control and severe colitis. (**A**) Integrated area under the bands representing protein, collagen, and glycoprotein. (**B**) Integrated area under the bands representing RNA. (**C**) Integrated area under the band representing DNA.

The regions of predominant protein (amide I and II), glycogen, collagen, and nucleic acids were quantitatively analysed as whole hyperspectral images (with each pixel representing the integrated area under the corresponding spectra), as shown in Figure 2. The same regions were then statistically analysed by comparing the integrated area under the average spectra for the entire image for each of the mucosal sections (Figure 3).

The colon sections displayed in the hyperspectral images (Figure 2) are representative sections acquired from control, mild, and severe colitis tissues. Corresponding H&E sections demonstrate mild colitis defined by mucosa-limited intrusion of the inflammatory infiltrate compared with severe colitis displaying marked transmural inflammatory cell infiltrate and extensive epithelial ulceration [22]. In the hyperspectral images, the highest and lowest intensity of the integrated regions of interest of the spectra are represented by the red and blue hues, respectively, as shown in the adjacent colour bars.

Amide I and II (Protein): The amide bands are the main protein bands in the spectra of biological materials. The integrated areas under the curve at 1700–1600 cm^−1^ (amide I) and 1580–1510 cm^−1^ (amide II) show increased quantities of protein with increasing severity of inflammation in the colon on both hyperspectral imaging and in comparisons of mean integrated areas under the curve for these bands (*p* < 0.000001).

Collagen: Collagen is represented in the integrated area under spectra over 1300–1200 cm^−1^, including maxima at 1280 cm^−1^, 1230 cm^−1^, and 1200 cm^−1^. Hyperspectral imaging shows that collagen does appear to be increased in inflamed mucosal sections, however, this did not reach statistical significance when comparing mean integrated areas under the curve (*p* = 0.15).

Glycogen: Glycogen has pure peaks located at 1151 cm^−1^, 1076 cm^−1^, and 1028 cm^−1^ in the spectra. Hyperspectral imaging over the integrated area under 1250–1000 cm^−1^, representing glycogen content, shows that glycogen is highest in control mucosa which contain glandular epithelia with mucin-producing goblet cells. Glycogen appears to be reduced with increasing inflammation, potentially due to goblet cell depletion, a commonly featured criteria in histological scoring systems of colitis severity along with erosions/ulceration with loss of epithelium. However, comparing mean integrated areas under the average spectra curve did not reach statistical significance (*p* = 0.57).

Nucleic acids: Hyperspectral imaging for RNA was performed by integrating the area under the spectra in the region 1120–1080 cm^−1^ which includes both the RNA specific band at 1115 cm^−1^ [23] as well as some nucleic acid phospholipid bands. Hyperspectral imaging for DNA was performed by integrating the area under the spectra in the region from 979–948 cm^−1^ containing the known absorbance for DNA at 960 cm^−1^. As these tissues have been fixed in formalin, most of the DNA would likely be in the dehydrated α-DNA form [17]. The results of both the mean integrated area for DNA (*p* < 0.002) and mean combined absorbance peaks for RNA (*p* < 0.000001) show that the greatest quantities of nucleic acids are found in severe colitis due to the increase in inflammatory cell infiltration.

Subsequently, colitis and control tissue display broad differences in biochemical composition in hyperspectral imaging, particularly in the regions of protein, DNA, and RNA which are increased with colitis. Hyperspectral imaging appears to also show increased collagen and reduced glycogen in inflamed mucosa, however, this did not correlate with statistical significance when comparing mean integrated areas under the spectral bands for these respective regions. The differences in the mean integrated area under the collagen and glycogen bands may be explained by sampling (as histologically graded severe colitis sections may still contain glandular epithelial mucosa) and the contribution of other components, particularly nucleic acids, to the absorbance at these wavenumber values.

#### 2.2.2. Artificial Neural Network Hyperspectral Image Classifier

**Figure 4:**
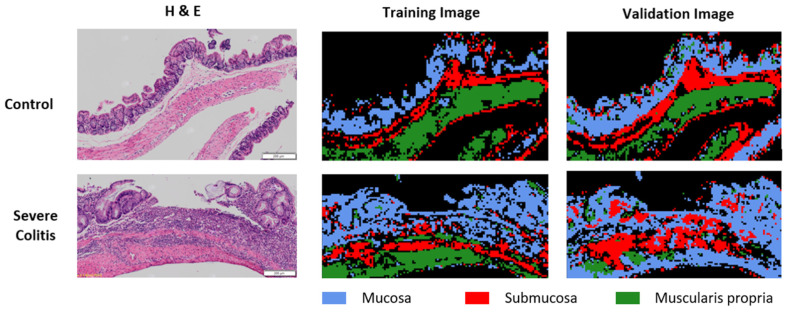
Hyperspectral image classification using ANN for controls and severe colitis. The training image depicts the classification performed on the entire original image using trained classes and the validation image uses trained classes from an independent sample.

Hyperspectral imaging classification using an artificial neural network (ANN) was performed on representative sections of controls and severe colitis. Figure 4 shows that using an ANN model (which was trained using only 10 spectra for each bowel layer) accurately predicted both the spectra contained in its own image as well as a completely independent test image (each containing up to 5000 untrained spectra). As the inflammatory infiltrate in severe colitis contains dense nucleated material, the spectra from distinct layers are more obscured and homogenous, which accounts for the image differences.

### 2.3. Tissue Colitis Classification

#### 2.3.1. Spectral Biomarkers for Colitis

A partial least squares discriminant analysis (PLS-DA) was performed using the pre-processed second derivative spectra from the region 1800–800 cm^−1^. This was performed on the spectra from the individual bowel layers (MC, SubMC, and MP) as well as all the layers combined.

The PLS-DA classification models using spectra from all bowel layers (five latent variables, LVs), the mucosa alone (five LVs) and the submucosa alone (five LVs) were highly accurate in classifying severe colitis and controls with cross-validated sensitivities and specificities of 91.4% and 93.3%, 92.5% and 99.8%, and 96.9% and 94.1%, respectively. The muscularis propria model (three LVs) had a slightly lower sensitivity of 85.8% and specificity of 92.9%, but this is not unexpected as the utilized dextran sodium sulfate (DSS) chemical colitis model does not typically result in transmural inflammation. The submucosa spectra appeared to provide the best discrimination between inflammation and normal tissue. The predominant wavenumber bands featured in the latent variables for the model incorporating all bowel wall layers were the protein bands in amide I, particularly the α-helix maxima at 1647 cm^−1^, but also other protein bands, such as 1481 cm^−1^, the collagen/nucleic acid band at 1230 cm^−1^, and the glycoprotein bands at 1076 cm^−1^ and 1030 cm^−1^.

**Figure 5:**
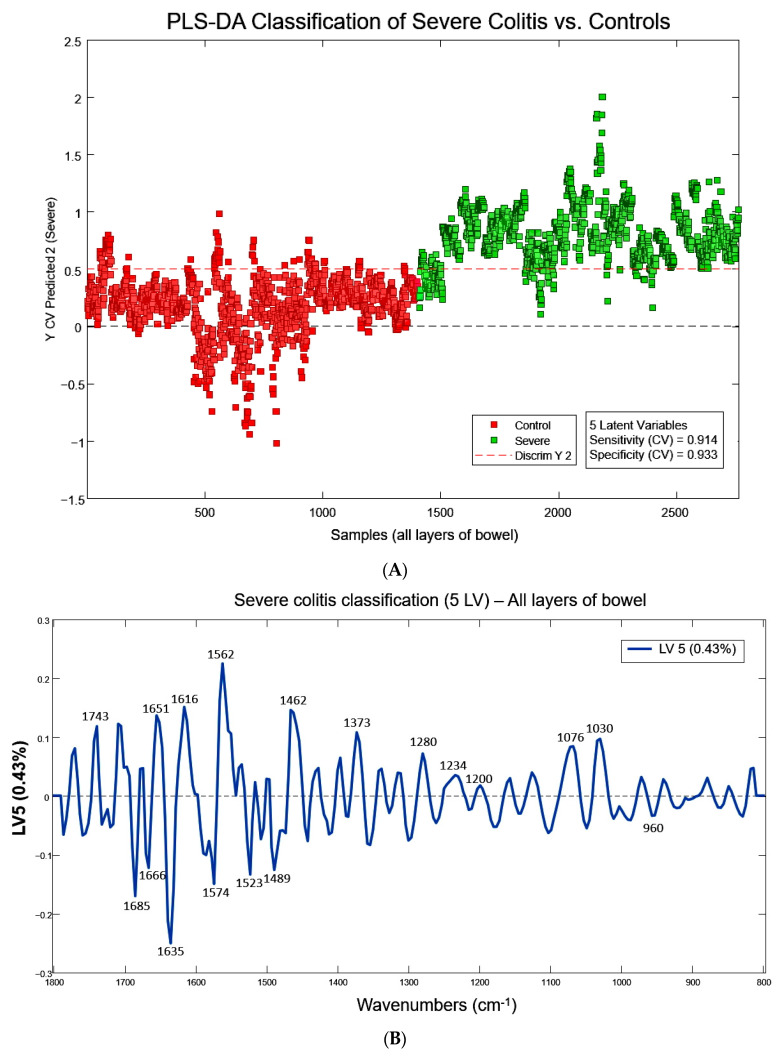
Partial least squares discriminant analysis model classifying spectra from all bowel wall layers as healthy tissue or severe colitis. (**A**) Classification of spectra from all layers of the bowel into controls and colitis. (**B**) All five latent variables included in the classification model for severe colitis.

Figure 5A shows the PLS-DA model using spectra from all the bowel wall layers and Figure 5B the summation of all five LVs used in the model which account for >99.5% of the variability with labelled wavenumber values of contribution and interest. The PLS-DA models for the individual bowel layers (MC, SubMC, MP) and the loadings from their respective LVs are found in the Supplementary Materials (Figures S1–S3).

**Figure 6:**
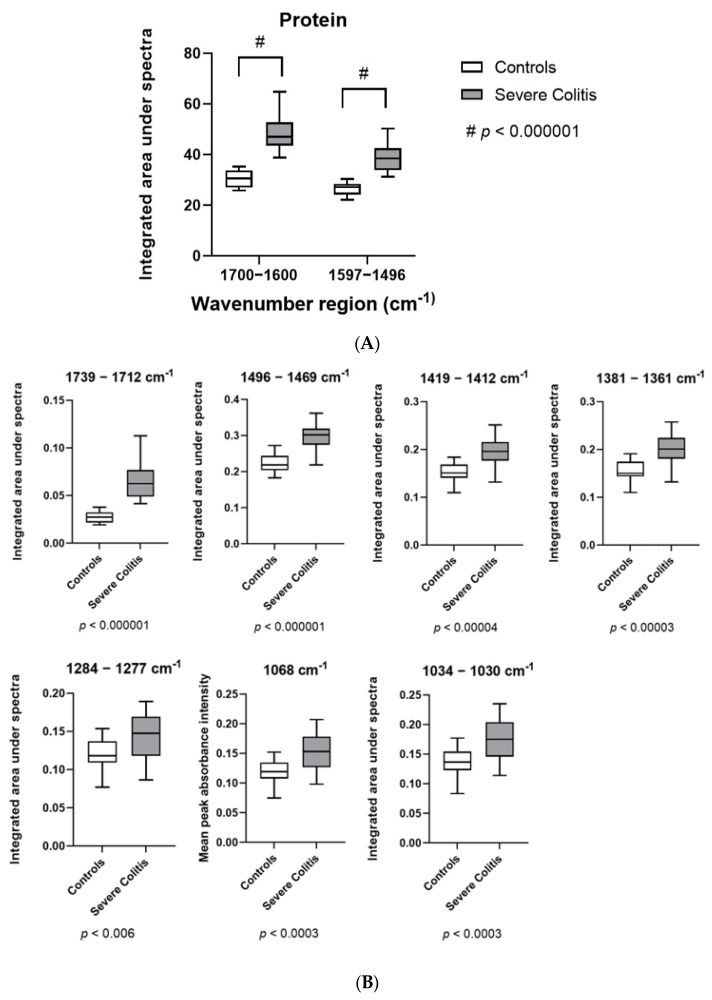
Significant VIP scores correlating to the PLS-DA classification model for healthy tissue and severe colitis from Figure 5. (**A**) Significant VIP scores representing amide I and II protein bands. (**B**) Other significant VIP scores, including those for glycoproteins and nucleic acids.

Quantitative analysis of biochemical composition corresponding to the significant variable importance in projection (VIP) scores for the PLS model revealed highly statistically significant differences between the control and the severe colitis groups, as shown in the box plots represented in Figure 6A,B. They largely correlate with the protein peaks at amide I (1700–1600 cm^−1^), amide II (1597–1496 cm^−1^), collagen (1284–1277 cm^−1^), glycoproteins, and nucleic acids (1068, 1034–1030 cm^−1^), which have remained consistent findings in our analyses.

#### 2.3.2. Colitis Severity Classification

**Figure 7:**
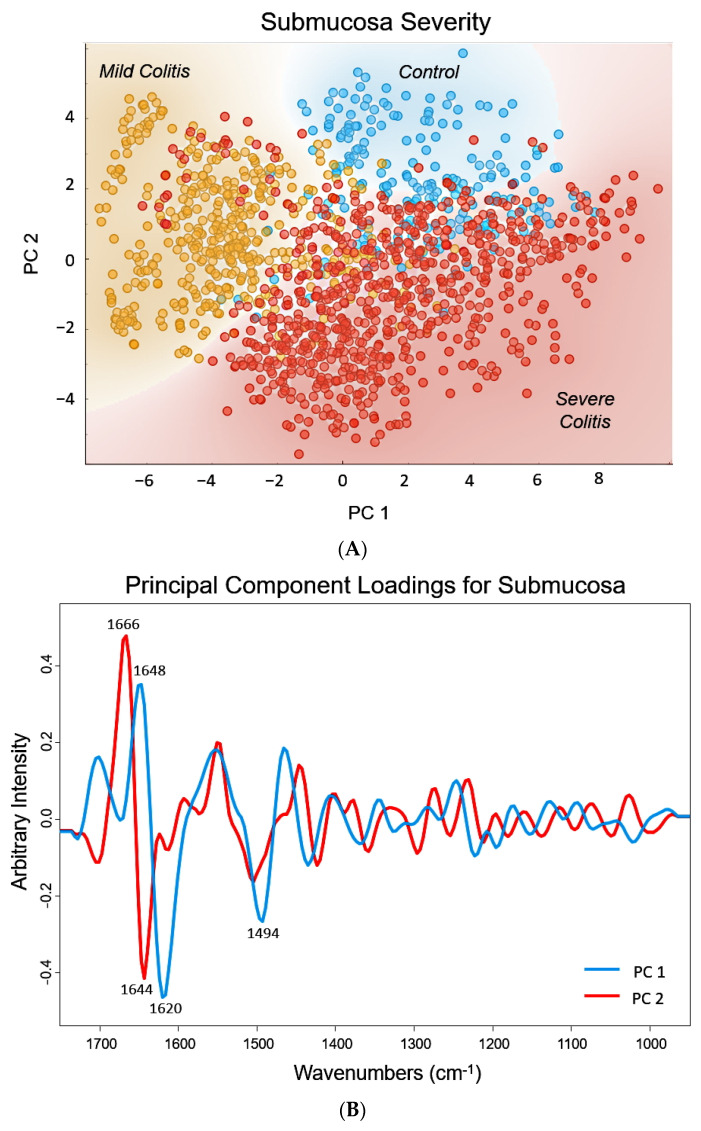
Unsupervised classification of colitis severity using the submucosa tissue spectra in a PCA model. (**A**) Plot of PC1 and PC2 using PCA from spectra obtained from the submucosa of healthy, mild, and severe colitis demonstrating separation between these classes. (**B**) Loadings plot for PC1 and PC2 demonstrating key features in the wavenumber bands that separate the classes.

Finally, a model was created to classify tissue spectra colitis severity into classes comprising controls, mild, moderate, and severe colitis. Firstly, unsupervised learning was undertaken using principal component analysis (PCA) of the pre-processed second derivative spectra. Figure 7A shows that controls, mild, and severe colitis spectra from the submucosa have accurately separated, largely due to the protein bands (1666, 1648, 1644, 1620, and 1494 cm^−1^) demonstrated by the maxima and minima for the principal component (PC) loadings for PC1 and PC2 on Figure 7B. The moderate colitis spectra are not shown in Figure 7 as its data lie over the mild/severe clusters and obscure the image.

PCA was also performed for variable dimension reduction prior to supervised machine-learning classifiers. The number of principal components (PCs) was chosen to account for at least 95% of the data and varied according to bowel layer. The remainder was thought to be largely due to noise. Several complex machine-learning classifiers were used for 10 stratified independent test sets, with the final results averaged for all sections of the bowel, altogether and individually (MC, SubMC, MP). The hyperparameters used in the individual classification models are listed in the Supplementary Materials (Table S1).

A total of 280 separate models were analysed. Overall, models using spectra from the submucosal section alone displayed the highest performance for severity classification based upon metrics for area under the receiver operating characteristic (AUROC) and classification accuracy (CA). From these submucosal classifiers, the best performing models for identifying each class of severity (controls, mild, moderate, and severe) were selected and stacked into an ensemble model which included an artificial neural network (ANN), support vector machines (SVM), random forest (RF), and k-nearest neighbour (kNN) algorithms. The average AUROC and CA (over 10 independent test sets) for the submucosal stack classification model was AUROC 0.95, CA 0.91 for controls, AUROC 0.88, CA 0.81 for mild colitis, AUROC 0.79, CA 0.78 for moderate colitis, and AUROC 0.85, CA 0.88 for severe colitis.

**Figure 8:**
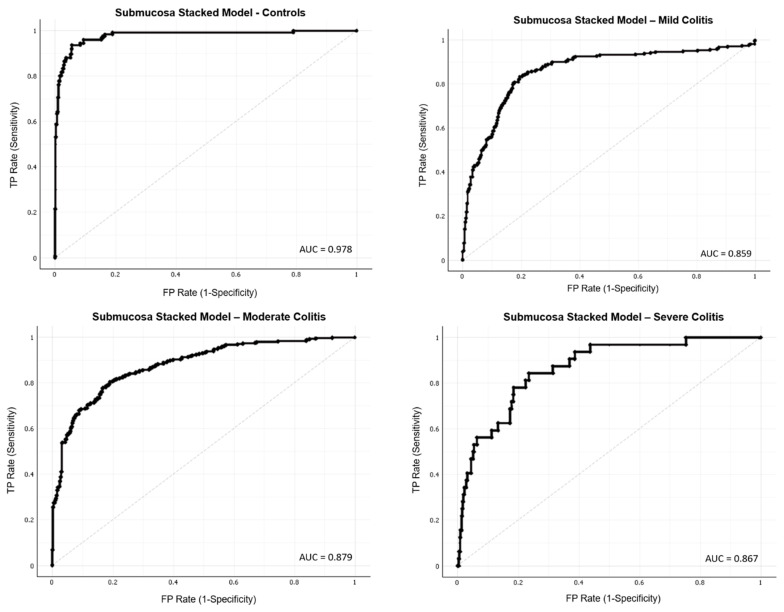
ROC and AUROC for stacked model classification performance according to colitis severity using submucosal spectra and validated by independent test sets. See Scheme 1 for more details.

An example of the receiver operating curves for the final resultant stacked submucosal model is shown in Figure 8.

## 3. Discussion

This study has characterised the tissue biochemical composition of colitis and its severity using infra-red microspectroscopy, demonstrating that colitis tissue exhibits increased quantities of protein, collagen, nucleic acids, but reduced glycogen when compared with normal colonic tissue. Our study also confirms that FTIR spectroscopy can accurately differentiate intestinal tissue samples of colitis from controls and classify the severity of inflammation based on the biochemical changes in tissue specimens. These results correspond with the histological scoring in a gold-standard murine model for IBD with a high degree of accuracy, particularly using spectra from the submucosal region for discrimination.

Lipid analysis has not been included as part of this biochemical compositional analysis using FTIR spectroscopy, as the formalin fixation process has likely affected tissue lipid composition and there are additional confounding bands from paraffin that obscure known lipid absorbance bands. Studies using unfixed tissue have identified the lipid band at ~1460 cm^−1^ (also found in paraffin) to be of potential significance when comparing colitis and colon cancer [10].

Our results highlight the potential of using FTIR spectroscopy to provide alternative biochemical information with significant advantages over conventional histology. While the technique we utilised does not necessarily provide individual molecular information, it does provide an entire biochemical overview of the sample and macromolecular information and has significant advantages. Infra-red spectroscopy preserves the sample, does not require sample preparation or staining, and removes the need for physical storage of specimens for over 10 years, as required in some countries. Some FTIR and Raman technologies allow for portable collection of spectra, with rapid acquisition in the order of minutes, allowing for utilisation as a potential point-of-care diagnostic with a potential application for use during gastrointestinal endoscopy. Furthermore, tissue spectra obtained with FTIR are reproducible and can avoid problems with subjective methods of assessment, including histological scoring with intrinsic issues of inter- and intra-observational errors.

To our knowledge, this study provides the only information utilising FTIR spectroscopy to characterise and classify the severity of colitis based on layers of the bowel, including the mucosa, submucosa, and muscularis propria. These findings are novel, as we have found that the degree of spectral differences between intestinal tissue types are more significant than those for varying degrees of inflammation within the same tissue type. Comparisons of our spectral results with the majority of published FTIR studies using intestinal tissue are difficult, as the focus has mainly been on differentiating colitis and colon cancer; the specimens are sufficiently variable to significantly affect spectral characteristics or are acquired via different modalities of spectroscopy, such as Raman, which cannot be directly compared. Subsequently, findings from the studies most relevant to this field are discussed below.

Lasche et al. characterised the spectra of individual human bowel wall layers in the context of colorectal adenocarcinoma [24]. While this is a different pathological condition, when assessing the constituents of the layers of the bowel, similar to our findings, the authors identified intense submucosal bands at 1235 cm^−1^ and 1281 cm^−1^, reflecting higher quantities of collagen in the submucosa, and dominating protein signals in amide I (1690–1620 cm^−1^) in the muscularis propria compared with the submucosa [24].

Katukuri et al. compared fresh colonic mucosal tissue from murine DSS colitis with controls [11]. They identified important absorbance bands at 1072 cm^−1^ (C-N stretch of glycoproteins), 1088 cm^−1^ (symmetric PO_2_^−^ stretch of nucleic acids), and 1740 cm^−1^ (C=O stretch of phospholipids) to distinguish inflammation from normal mucosa with 92% sensitivity and 83% specificity in a PLS model [11]. While the influence of the protein bands from amides I and II had a much greater influence on our mucosal PLS-DA model for distinguishing severe colitis from healthy controls (see Supplementary Materials, Figure S1B), when analysing wavenumber bands for significant VIP scores, the same phospholipid band at 1739 cm^−1^ and glycoprotein band at 1072 cm^−1^ also featured. We also found that the collagen band at 1280 cm^−1^ contributed to significant VIP scores. However, it is difficult to directly compare our study results due to variations in sample morphology (full thickness FFPE specimens vs. fresh mucosal tissue only) and their study did not explore DSS colitis severity.

Although our results cannot be directly compared with inflammatory spectral biomarkers from Raman spectroscopy, biochemical differences in IBD have also been confirmed using this technique. Unlike infra-red spectroscopy, Raman spectroscopy uses laser energy to measure non-elastic light scattering to produce biochemical information. It has been shown to distinguish tissue [25] and plasma samples [26] from IBD and healthy controls, Crohn’s disease from ulcerative colitis [27,28], as well as active inflammation from mucosal healing based on varying profiles in lipids, phosphatidylcholines, myoglobin, and carotenoids [29,30,31]. Fibreoptic Raman probes have also been developed with the potential for application in point-of-care endoscopy, as the spectra acquired are not obscured by the presence of water in the colon [32].

Finally, spectral interpretation can be difficult, particularly in complex biological specimens, such as tissues. Multiple molecules in tissue may contribute to the infra-red absorbance intensity at a specific wavenumber value and differences in the spectral waveforms for controls and colitis in tissue spectra appear subtle to the naked eye. In this study, PCA demonstrated that the degree of variation amongst the bowel layers (and subsequently different types of tissue, e.g., epithelium, extracellular matrix, and muscle) was greater than the variation between colitis and controls in each respective bowel wall layer. Subsequently, there is a need to use more sophisticated classifiers, such as those in machine learning, to assist in assigning classes of biological spectra. However, it is important to confirm that the features selected, in this case the wavenumber values, contain valid biological information rather than noise. This study has shown through multiple analytical modalities, including integrated areas under the spectral curve and assessing LV and VIP scores in the PLS-DA model, that the differences separating controls and the classes of colitis are consistent and biologically plausible. While sampling error remains a source of potential bias, this also applies to histopathological specimens.

Nonetheless, this study presents promising applications for biospectroscopy in IBD. Further studies are required using this technology to provide biochemical assessments for diagnosis and activity assessment of human Crohn’s and ulcerative samples correlating with validated endoscopic and histological scoring systems and clinical outcomes. Concurrent paired assessments of other biospecimens, including serum or stool specimens, may allow for an even less invasive method of colitis diagnosis and assessment. A pilot study used surface-enhanced Raman scattering to differentiate spectra from the serum of UC and healthy individuals with a sensitivity of 89% and specificity of 94% [33]. FTIR serum studies of murine colitis models demonstrate that spectroscopy can detect differences in control and colitis groups [12] which normalise upon treatment with anti-tumour necrosis factor alpha [13]. Furthermore, previous studies have consistently demonstrated that FTIR spectroscopy can differentiate cancer, colitis, and normal tissue [8,9,10] with sensitivities of 81–98% and specificities of 70–93%. This suggests that FTIR spectroscopy may also be utilised for dysplasia surveillance in IBD, as it can distinguish inflammation from malignancy.

## 4. Materials and Methods

### 4.1. Colitis Induction and Treatment Intervention

Intestinal samples were obtained from animal experiments that were approved by the Monash University Animal Ethics Committee (AE#B12/02, 9 April 2015) and conducted in accordance with the Australian Code of Practice for the Care and Use of Animals for Scientific Purposes (2006). Chronic colitis was induced in 8-week-old C57/BL6 mice with 3 cycles of 1.5% (weight/volume) dextran sodium sulfate (DSS) via the oral route (*n* = 27) over 9 weeks. Half of the murine cohort of DSS colitis (*n* = 14) were treated with weekly intravenous human amnion epithelial cells at a dose of 2 × 106 cells for 4 weeks. This intervention was shown to reduce histological and immunochemical severity of intestinal inflammation and fibrosis [34].

### 4.2. Sample Preparation and Assessment

FFPE murine colon tissue sections were cut in 5 μm sections and placed on a reflective slide (Kevley Technologies, Chesterland, OH, USA) then de-paraffinised with xylene. An adjacent 5 μm tissue section was collected and stained with haematoxylin and eosin (H&E) for histological comparison. Corroborative histological assessment of colitis severity was conducted on the adjacent H&E slide using criteria with specific scoring features for chemically induced colonic inflammation [22].

### 4.3. FTIR Hyperspectral Imaging Acquisition

For each mouse, 3 separate colitis sections were imaged as technical triplicates, and for control mice, 6 different sections were imaged. Sections included the most and least severely inflamed areas selected via histological assessment. Specific regions of interest were obtained both collectively as an entire section and separately from anatomical layers of the bowel—mucosa (MC), submucosa (SubMC), and muscularis propria (MP)—to reduce sampling bias due to variable quantities of bowel wall layers. Images were acquired using the Agilent Cary 670 FPA-IR coupled with the Agilent Cary 620 microscope in transflection mode with a 15× microscope lens objective. To achieve a high signal-to-noise ratio, 128 and 64 coadded scans were collected in each measurement for the background and tissue, respectively, in the wavenumber region 1900–700 cm-1 using a spectral resolution of 8 cm^−1^ and 2 × 2 pixel aggregation giving a theoretical pixel size of 11 µm2.

### 4.4. Spectral Data Preprocessing

Data sampling, pre-processing, and analysis was undertaken with the licensed software packages MATLAB (MathWorks, Natick, MA, USA), CytoSpec version 2.00.06 (Berlin, Germany), Unscrambler version 11.0 (Camo Analytics, Oslo, Norway), Quasar 0.9.0 (Orange-Spectroscopy [35]), and PLS Toolbox (Eigenvector Research Incorporated, Washington, DC, USA). Spectral waveform image preparation was carried out using OPUS version 8.0 (Bruker, Ettlingen, Germany).

Analyses was undertaken using the spectral range 1801–798 cm^−1^, designated the “biological fingerprint” region, as no obvious biomolecule absorbance external to this range was apparent. Data preprocessing and outlier detection is required to improve analysis and classification model performance [36]. This included removal of background spectra, rubber band correction of the baseline for analyses that required integration over regions of interest, and transformation to second derivative spectra using a Savitzky–Golay smoothing function with nine points to a third-order polynomial and normalised using the standard normal variate over the entire region for classification. The Savitzky–Golay algorithm reduces random noise in the data, and normalization with SNV reduces multiplication interference, slope variation, and scatter effects in the sample [10]. The entire dataset (>500,000 spectra) was then averaged to approximately 25 spectra per sample to ensure a balanced dataset, ease of data management, and reduce computation power requirements.

### 4.5. Spectral Data Analysis

**Scheme 1:**
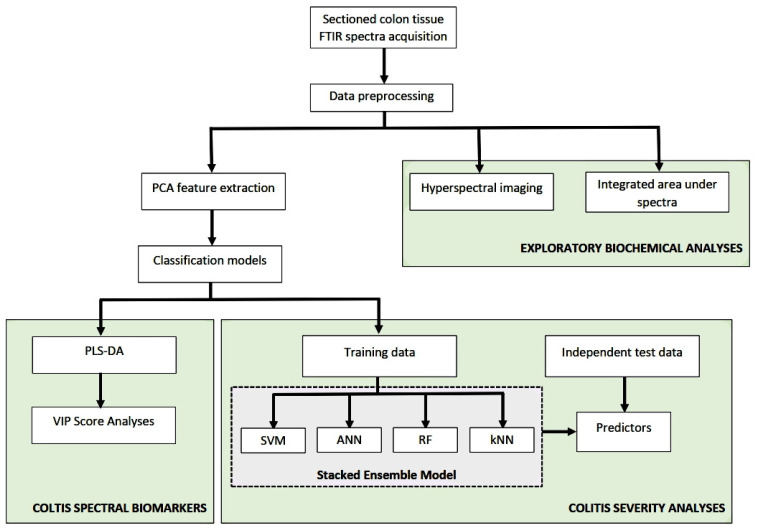
Spectral data analyses workflow.

Scheme 1 demonstrates the data analysis work flow used for this project.

Whole hyperspectral image analyses of the tissue sections were quantitatively assessed by integration of the area under the spectra representing broad components of protein, glycogen, nucleic acids, and collagen. Multiple unpaired *t*-tests using Welch correction for unequal variances were performed using GraphPad Prism version 9.0.0 for Windows (GraphPad Software, San Diego, CA, USA) for comparison of peak absorbance intensities and integrated areas under the spectra curve when comparing severe colitis and control mucosa.

Hyperspectral image classification was performed using a three-layer artificial neural network (ANN) with a single hidden layer accessed through the Stuttgart Neural Network Simulator (University of Stuttgart) and incorporated into CytoSpec version 2.00.06. Supervised training of classes from MC, SubMC, and MP from control and severe colitis sections was performed using only 10 spectra per class at 100 iterations to classify each training image and an independent validation image.

A partial least squares discriminant analysis (PLS-DA) was performed using second-derivative spectra, including all bowel wall layers as well as separate sub-analyses, using spectra from the MC, SubMC, and MP in both control and severe colitis samples. This was performed to identify potential colitis biomarkers by analysing the contributions from the latent variables (LVs) and variable importance in projection (VIP) scores, which were considered significant if greater than one. The numbers of LVs chosen were based on selecting the minima values in the latent variable versus cross-validation error plots. Cross-validation was performed using a venetian blind method. All wavenumber values with a significant VIP score were further evaluated using multiple unpaired *t*-tests with Welch correction to confirm that there was a statistically significant difference between the controls and severe colitis at the averaged peak absorbance intensity per sample (for discrete wavenumber values) or integrated area under the spectra (if the series of wavenumber values correlated to a maxima).

Model classifiers for comparing colitis severity were created using machine-learning algorithms chosen to suit multi-class classification [37]. Unsupervised analysis was undertaken with principal component analysis (PCA), with the number of components chosen to reflect at least 95% of the data; the details are discussed individually with models. PCA was employed to explore features used for classification as well as for dimension reduction. Supervised classification was performed using support vector machines (SVMs), artificial neural networks (ANNs), random forests (RFs) and k-nearest neighbours (kNNs) for the development of a classification model for colitis severity. The highest performing models were stacked in an ensemble to improve performance. The hyperparameters used for individual machine-learning classifiers are listed in Supplementary Table S1 in the Supplementary Materials. To prevent model overfitting, cross-validation was performed using both stratified nine-fold cross-validation, initially, and later a randomized stratified independent test set partitioned at 70% training and 30% testing on shuffled data. To reduce sampling bias from this method, the modelling was repeated 10 times on 10 separate hold-out partitions, with the average of the resulting evaluation metrics recorded. The distribution of the classes for the 10 partitions for training and testing are shown in Supplementary Figure S4 in the Supplementary Materials.

## 5. Conclusions

Our study confirms that the biochemical changes in colitis as detected by FTIR spectroscopy are distinct compared with healthy controls and that, with multivariate analyses using machine-learning algorithms, the biochemical data can be interpreted for colitis severity assessment. This proof-of-concept study shows that FTIR spectroscopy has significant potential to assess inflammatory conditions, such as IBD, using a method that can be standardized and automated with potential application as a point-of-care diagnostic technique.

## Figures and Tables

**Table 1:** Infra-red band assignments for intestinal tissue.

Wavenumber Values (cm^−1^)	Band Assignment
~1743	Ester carbonyl (lipid) [10]
~1685	Amide I anti-parallel β-pleated sheet protein secondary structure [12]
~1646	Amide I α-helix protein secondary structure [10]
~1639	Amide I β-pleated sheet protein secondary structure [15]
~1539	Amide II β-pleated sheet [15]
~1515	Amide II [16]
~1450	Carboxylate group (proteins) [17]
~1390	Lipids [16]
~1310	Amide III [10]
~1280	Collagen [15]
~1230	Collagen, nucleic acids [15]
~1200	Collagen [15]
~1151	Carbohydrates and glycogen [18]
~1115	RNA [12]
~1076	Mannose and glycoproteins, nucleic acids [12]
~1053	Glycogen and other carbohydrates [16]
~1028	Glycoproteins including glycogen [19]
~999	RNA [17]
~964	Deoxyribose (mainly DNA) [8]

## Data Availability

The data presented in this study are available on request from the corresponding author. The data are not publicly available due to intellectual property arising from the use of cell therapy.

## Supplementary Materials

The following supporting information can be downloaded at: https://www.mdpi.com/article/10.3390/ijms23052849/s1.

## Abbreviations

ANN	Artificial neural network
AUROC	Area under receiver operating characteristics
CA	Classification accuracy
DSS	Dextran sodium sulfate
FTIR	Fourier-transform infra-red
H&E	Haematoxylin and eosin stain
IBD	Inflammatory bowel disease
kNN	k-nearest neighbour
LV	Latent variables
MC	Mucosa
SubMC	Submucosa
MP	Muscularis propria
PC	Principal components
PCA	Principal component analysis
PLS-DA	Partial least squares discriminant analysis
