# Supplementary material for: Fourier-Transform Infra-Red Microspectroscopy Can Accurately Diagnose Colitis and Assess Severity of Inflammation
Source: Int J Mol Sci. 2022 Mar 5;23(5):2849. doi: 10.3390/ijms23052849 (PMC8911059; doi:10.3390/ijms23052849)
Supplement: Supplementary file 1 [file ijms-23-02849-s001.zip › ijms-1602710-supplementary.pdf]

Supplementary Data

Supplementary Figure S1A. Partial least squares discriminant analysis model classifying bowel mucosal spectra for severe colitis compared with controls

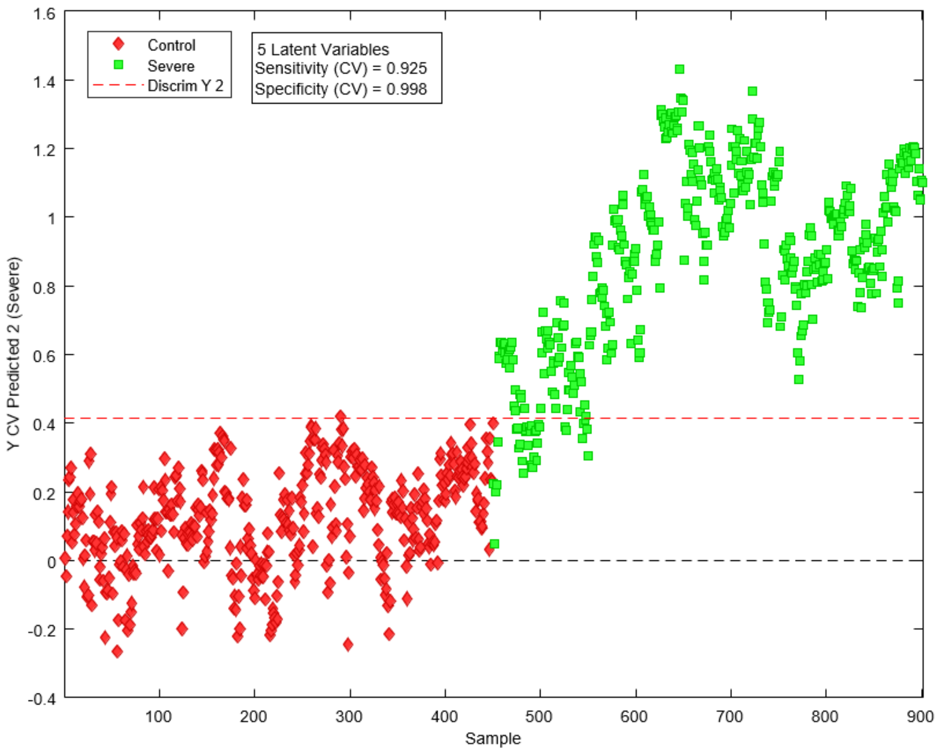

Supplementary Figure S1B. All 5 latent variables included in the mucosal model classification for severe colitis

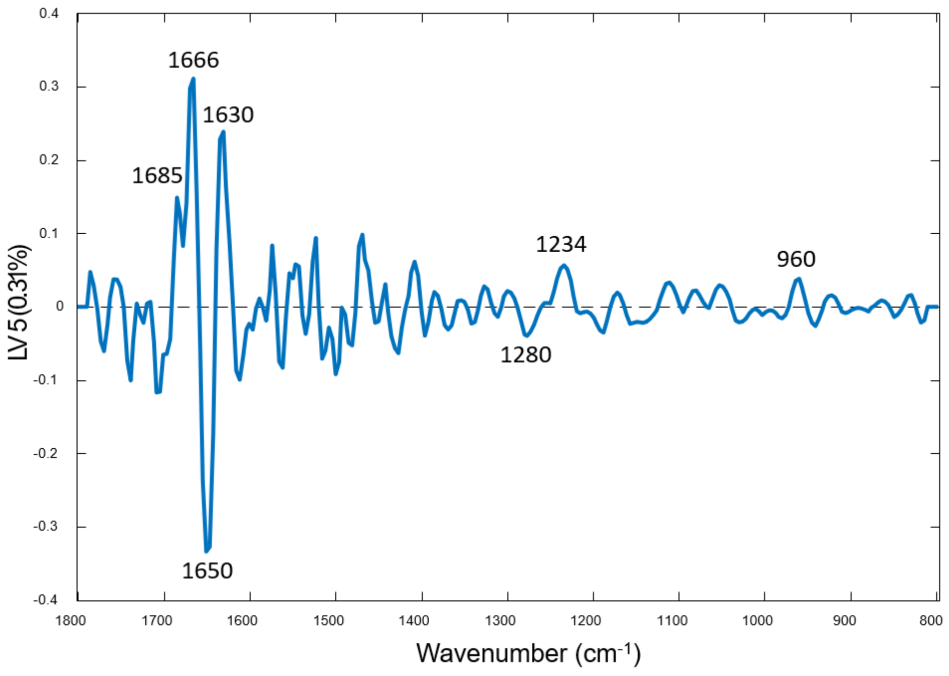

Supplementary Figure S2A. Partial least squares discriminant analysis model classifying submucosa spectra for severe colitis compared with controls

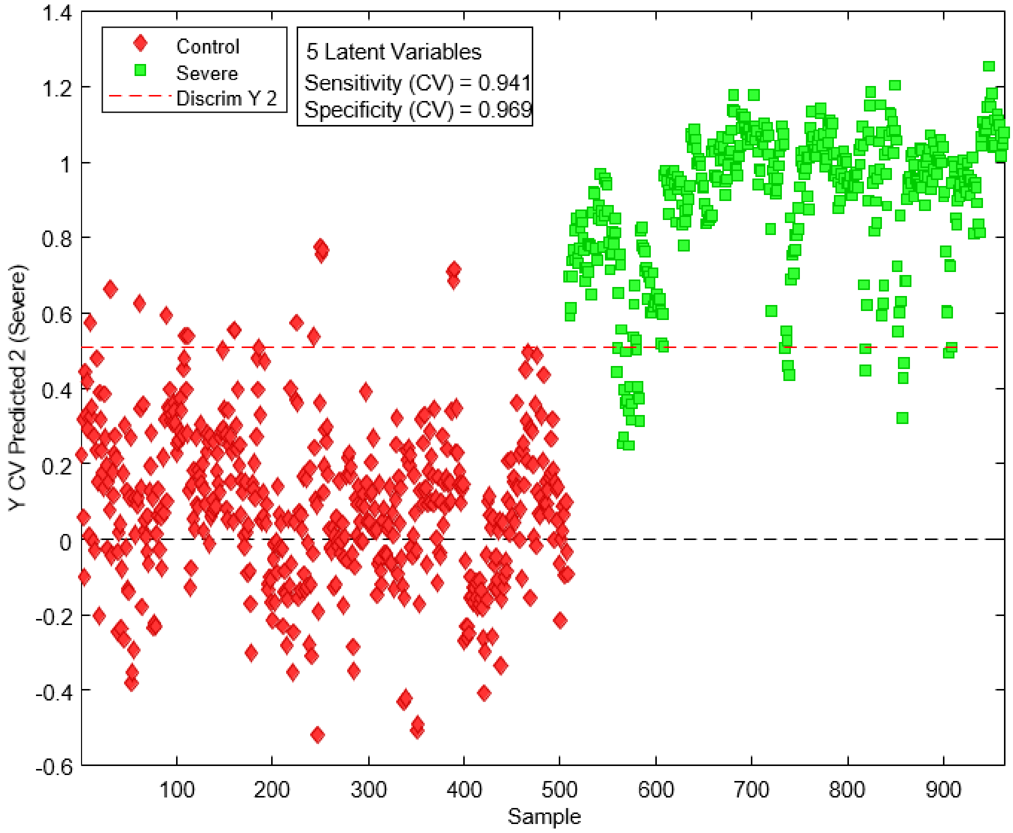

Supplementary Figure S2B. All 5 latent variables included in the submucosal classification model for severe colitis

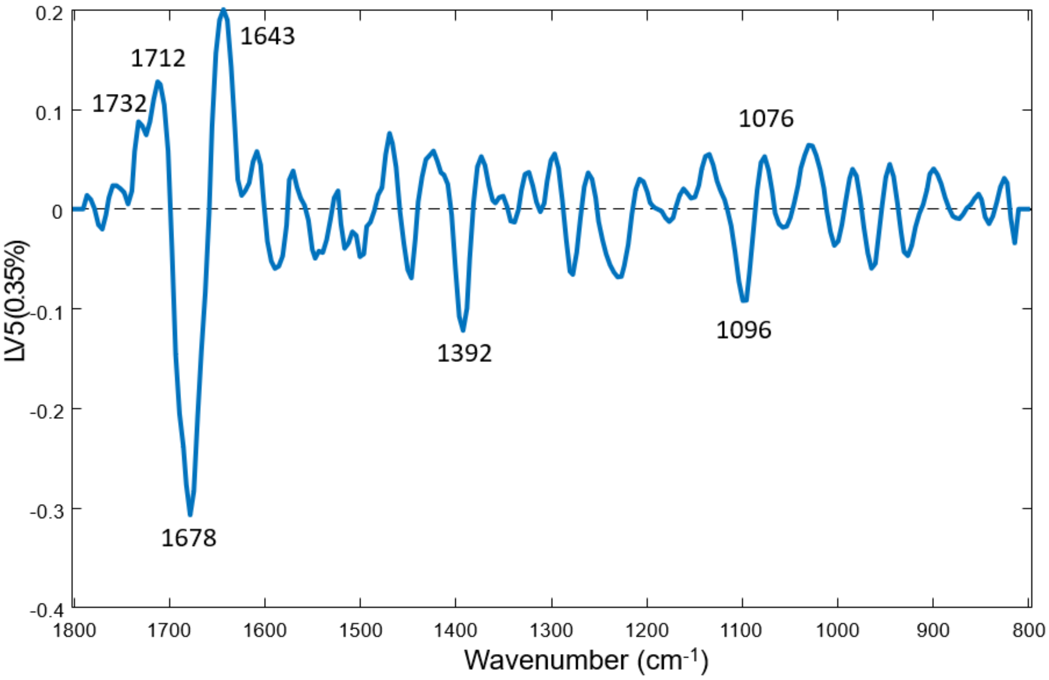

Supplementary Figure S3A. Partial least squares discriminant analysis model classifying muscularis propria spectra for severe colitis compared with controls

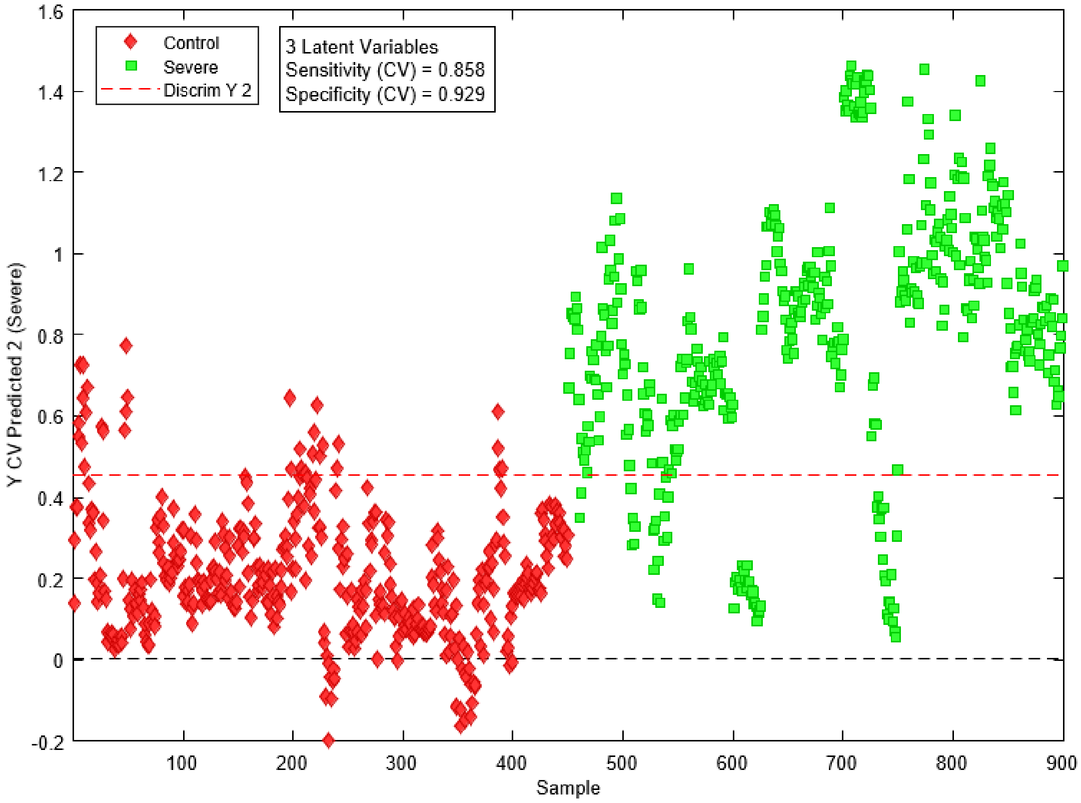

Supplementary Figure S3B. All 3 latent variables included in the muscularis propria classification model for severe colitis

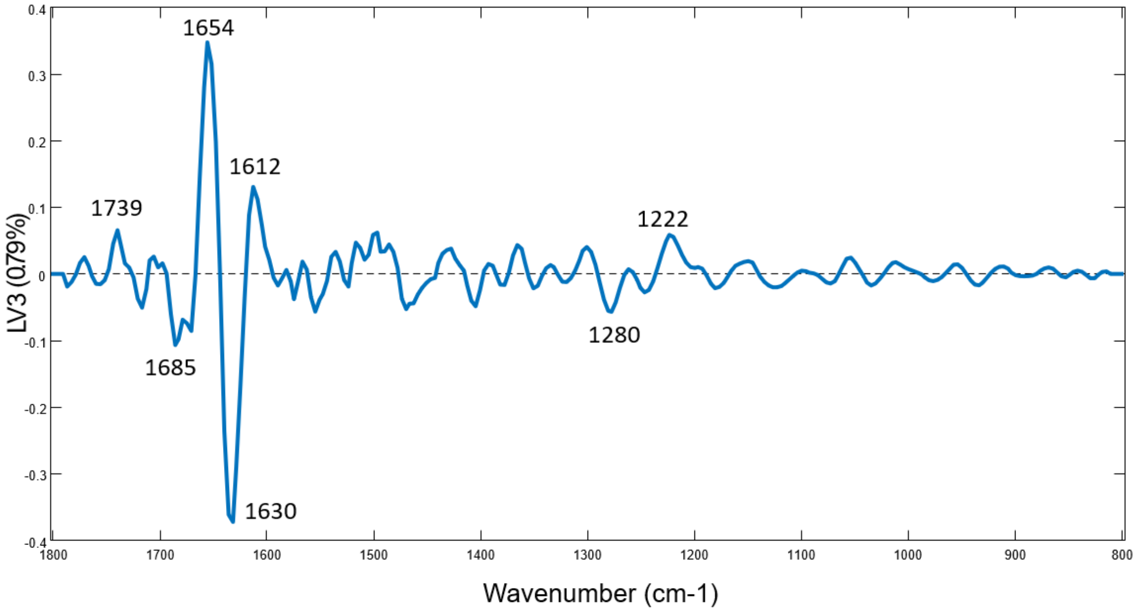

Supplementary Figure S4. Hold-out partitions at 70% training, 30% testing stratified for severity of colitis

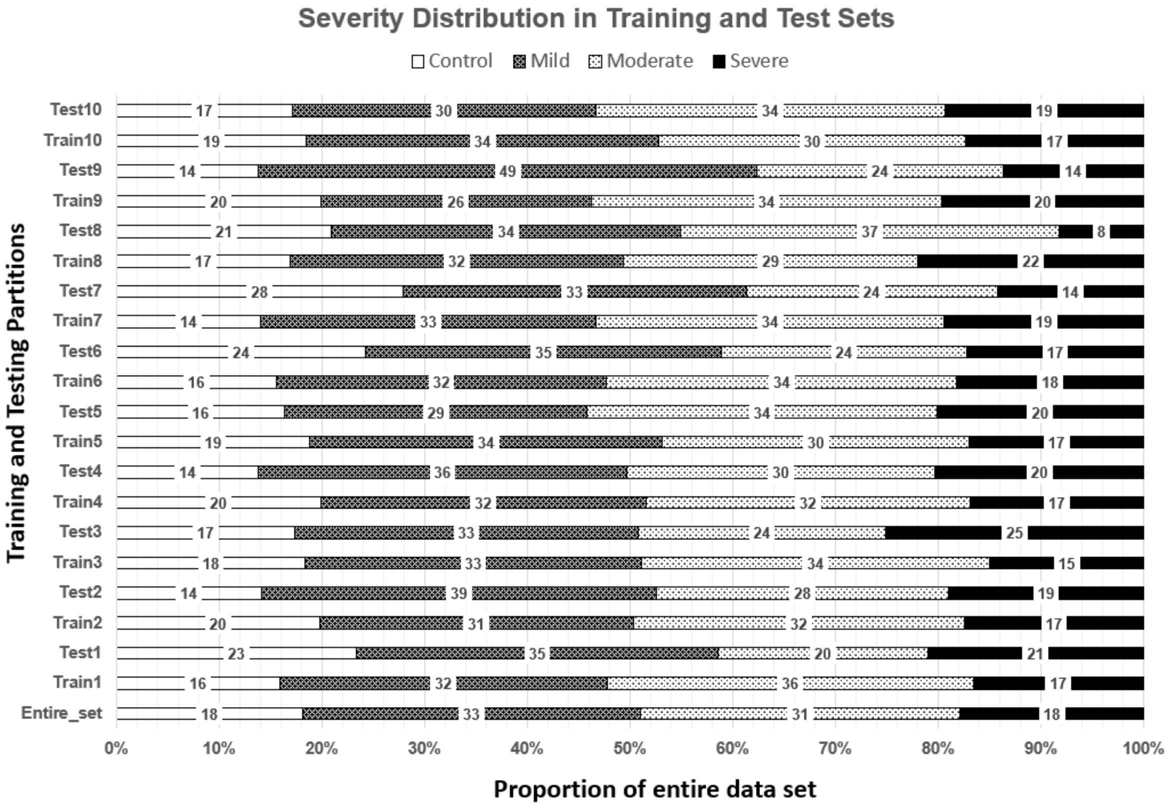

Supplementary Table S1. Hyperparameters for machine learning models

Bowel layer	No. of PCs (% data)	Classifier	Model parameter settings
**All layers**	15 (95%)	kNN	5 nearest neighbours, Euclidean, uniform weight
		RF	10 trees, replicable training, subsets < 5 not split
		SVM	RBF kernel, cost 10.0, gamma 0.01, 100 iterations
		ANN	Nodes in layers (15, 8, 6, 4), rectified linear activation function (ReLu), Adam solver, L2 regularisation 0.0001, 500 iterations
		LR	L1 Lasso regularisation, C parameter = 1
**MC**	13 (97%)	kNN	5 nearest neighbours, Euclidean, uniform weight
		RF	10 trees, replicable training, subsets < 5 not split
		SVM	RBF kernel, cost 10.0, gamma 0.01, 100 iterations
		ANN	Nodes in layers (13, 8, 6, 4), rectified linear activation function (ReLu), Adam solver, L2 regularisation 0.0001, 500 iterations
		LR	L1 Lasso regularisation, C parameter = 1
**SubMC**	22 (95%)	kNN	5 nearest neighbours, Euclidean, uniform weight
		RF	10 trees, replicable training, subsets < 5 not split
		SVM	RBF kernel, cost 10.0, gamma 0.01, 100 iterations
		ANN	Nodes in layers (22, 8, 6, 4), rectified linear activation function (ReLu), Adam solver, L2 regularisation 0.0001, 500 iterations
		LR	L1 Lasso regularisation, C parameter = 1
**MP**	10 (97%)	kNN	5 nearest neighbours, Euclidean, uniform weight
		RF	10 trees, replicable training, subsets < 5 not split
		SVM	RBF kernel, cost 10.0, gamma 0.01, 100 iterations
		ANN	Nodes in layers (10, 8, 6, 4), rectified linear activation function (ReLu), Adam solver, L2 regularisation 0.0001, 500 iterations
		LR	L1 Lasso regularisation, C parameter = 1

kNN k-Nearest Neighbour, RF Random Forest, SVM Support Vector Machines, ANN Artificial Neural Network, LR Logistic Regression
